# Persistence of F-Specific RNA Coliphages in Surface Waters from a Produce Production Region along the Central Coast of California

**DOI:** 10.1371/journal.pone.0146623

**Published:** 2016-01-19

**Authors:** Subbarao V. Ravva, Chester Z. Sarreal

**Affiliations:** Produce Safety and Microbiology Research Unit, U.S. Department of Agriculture, Agriculture Research Service, Western Regional Research Center, Albany, California, United States of America; Catalan Institute for Water Research (ICRA), SPAIN

## Abstract

F+ RNA coliphages (FRNA) are used to source-track fecal contamination and as surrogates for enteric pathogen persistence in the environment. However, the environmental persistence of FRNA is not clearly understood and necessitates the evaluation of the survival of prototype and environmental isolates of FRNA representing all four genogroups in surface waters from the central coast of California. Water temperature played a significant role in persistence–all prototype and environmental strains survived significantly longer at 10°C compared to 25°C. Similarly, the availability of host bacterium was found to be critical in FRNA survival. In the absence of *E*. *coli* F_amp_, all prototypes of FRNA disappeared rapidly with a D-value (days for one log reduction) of <1.2 d from water samples incubated at 25°C; the longest surviving prototype was SP. However, in the presence of the host, the order of persistence at 25°C was QB>MS2>SP>GA and at 10°C it was QB = MS2>GA>SP. Significant differences in survival were observed between prototypes and environmental isolates of FRNA. While most environmental isolates disappeared rapidly at 25°C and in the absence of the host, members of genogroups GIII and GI persisted longer with the host compared to members of GII and GIV. Consequentially, FRNA based source tracking methods can be used to detect phages from recent fecal contamination along with those that persist longer in the environment as a result of cooler temperatures and increased host presence.

## Introduction

Fecal contamination of ground and surface waters can cause significant environmental and health hazards. Tracking the sources of contamination is critical in designing pollution prevention and pathogen control measures. Several chemicals and biomarkers were used to track contamination resulting from human or animal sources [1–3]. Of these indicators, F+ RNA coliphages (FRNA) were often used as source identifiers to track fecal contamination in ground and surface waters [4, 5], produce [6], estuarine oysters [3], and as surrogates for enteric viral pathogens [7–9]. Using FRNA, contamination can be sourced to either animals or humans based on the association of four different genogroups with specific hosts. Genogroups I and IV (GI and GIV) are generally associated with animals, while genogroups II and III (GII and GIII) are associated with humans [10, 11]. Thus, human fecal pollution of river and ground waters was predicted based on a high abundance of phages from GII [5, 12, 13]. In a recent study, however, species of GIII predominated in both river and creek waters suspected of human activities nearby [14]. Likewise, contamination of surface waters from nearby farms was linked with the high prevalence of members of GI and GIV [15]. In addition to being source identifiers, some FRNA have shown preferential distribution across geographic distances. Members of GII were found to be prevalent in mainland Japan, whereas phages of GIII were isolated from the southern part of Japan and Southeast Asia (Taiwan, the Philippines, Singapore, and Indonesia) [16, 17].

Although beneficial as fecal indicators, differences in survival characteristics amongst FRNA species make it difficult to discern their proportions in environmental samples. Based on the limited data, members of GI and GII were known to persist longer in the environment [18, 19] and resist disinfection treatments compared to GIII and GIV [20]. In a separate study, phage isolates of GI have shown the highest resistance to environmental stresses and inactivation processes, followed by GII, GIII and GIV [18]. In contrast, the persistence of FRNA of different genogroups varied under different conditions, but temperature and pH were suggested to be major factors responsible for their persistence in river water [21]. At 4°C, phages of GI and GII were detectable even after 110 days, while GIII and GIV were reduced to detection limits after 3 weeks and 10 days, respectively. All of them disappeared rapidly at 20°C [19]. In a separate study, temperature correlated significantly with the decay rate of MS2, a prototype of GI, in ground water [22]. FRNA are known also to persist longer in biofilms compared to wastewater [23]. In addition, environmental FRNA differed in persistence compared to prototypes [24]. Thus, the differences in survival, as influenced by the environmental conditions, seem to alter the prevalence and proportion of FRNA.

In a recent study, we attempted to model source tracking shiga-toxigenic *Escherchia coli* and *E*. *coli* O157:H7 using generic *E*. *coli*, coliform bacteria, F-specific DNA coliphages and FRNA [14]. Owing to their scarcity, shiga-toxigenic *E*. *coli* could not be correlated with the prevalence of FRNA in river and creek water. In addition, higher prevalence of MS2-like phages (GI) was expected to signify the presence of cattle upstream from sampling sites. Instead, QB-like phages (GIII) were detected to indicate possible human fecal pollution. Since these observations were contrary to previous reports [18, 20], studies were conducted comparing environmental FRNA persistence to prototypes in surface water samples from the produce production region of the central coast of California. Since the availability of a bacterial host is critical for phage survival, persistence tests compared the survival of phages in water samples with or without added host bacteria. In addition, we noticed seasonality in the prevalence of FRNA in surface waters [14]; therefore, persistence measurements also included temperature effects. Survival of prototypes was monitored in waters at two different temperatures representing average summer and winter temperatures for the region. Our results demonstrate the suitability of FRNA to source-track fecal contamination in surface waters.

## Materials and Methods

### Ethics statement

Water samples were from Gabilan Creek near Salinas, California. Special permits are not required for water collection from this publicly accessible place.

### Surface waters

Water was collected from Gabilan Creek at Old Stage Road (GPS co-ordinates: 36.78028084N and -121.5848696W) near the city of Salinas. Surface water from the creek was collected using a bottle attached to a telescopic pole and transported on ice to the laboratory. Water samples were refrigerated for <18 h prior to use in phage persistence studies. The water samples were analyzed for total FRNA and for chemical composition (Table 1).

**Table 1:** Chemical composition of surface waters used.

		Date of water collection				
Chemical component^a^	Units	5-13-15	5-26-15	6-3-15	6-17-15	7-20-15
Na	meq/L	1.43	1.48	1.48	1.48	1.52
Ca	meq/L	6.49	5.99	5.74	6.04	6.54
Mg	meq/L	2.14	2.06	2.14	2.14	2.22
HCO_3_	meq/L	5.28	4.89	4.69	5.16	5.38
Cl	meq/L	1.80	1.44	1.44	1.52	1.58
Fe	mg/L	0.04	0.04	<0.04	<0.04	<0.04
Mn	mg/L	<0.01	<0.01	<0.01	<0.01	<0.01
P	mg/L	0.07	0.09	0.10	0.10	0.16
K	mg/L	1.4	1.3	1.3	1.3	1.6
NO_3_	mg/L	<2	<2	<2	<2	2
SO_4_	mg/L	89	87	88	88	93
B	mg/L	0.05	0.06	0.07	0.06	0.07
Dissolved solids	mg/L	666	616	601	639	674
Adjusted SAR^b^	mg/L	0.96	1.00	0.99	1.00	1.01
TSS^c^	mg/L	56	288	92	92	40
Turbidity	NTU	0.32	0.69	0.32	0.52	0.40
EC^d^	dS/m	0.94	0.90	0.89	0.92	0.95
pH		8.1	8.1	8.1	7.9	8.2
Strains assayed		MS2	QB	SP	GA	All environmental

^a^ Analysis performed by A & L Western Agricultural Laboratories, Inc, Modesto, CA

^b^ SAR: sodium adsorption ratio

^c^ TSS: total suspended solids

^d^ EC: electrical conductivity.

### Cultures of FRNA

Both prototype and environmental strains of FRNA were evaluated for survival in surface waters. Prototype strains MS2, GA, QB and SP representing genogroups GI, GII, GIII and GIV, respectively, were gifts from Prof. M. Sobsey of the University of North Carolina. Phages previously [14] isolated from surface waters from a produce production region of California (Table 2) representing all four genogroups were included to compare persistence of phages native to these waters to prototypes. Phages were grown in tryptic soy broth (TSB) supplemented with ampicillin/streptomycin (15 μg/mL) and log-phase growth of *E*. *coli* F_amp_ (*E*. *coli* HS(pF_amp_)R, ATCC 700891, American Type Culture Collection, Manassas, VA) as described in US EPA method 1601 [25]. Bacterial host cells were initially grown overnight in TSB with ampicillin/streptomycin at 37°C and used as inoculum to prepare fresh batch of log-phase growth. Each FRNA inoculated in 25 mL of log-phase host cell growth was incubated overnight at 37°C to promote phage growth and centrifuged at 10,000 x *g* for 10 min to remove host cells. The supernatant containing the phage was filtered through 0.2 μm filter and stored at 10°C. The phage preparations were used within 24 h for persistence studies. Concentration of these stocks was 8.7 ± 0.6 log PFU/mL.

**Table 2:** Environmental strains used.

Strain	Genogroup	Source^a^	Date of sampling^b^
GI-1	GI	ALICAR	1-2-2013
GII-1	GII	ALICAR	1-2-2013
GII-2	GII	RECVIC	1-15-2013
GIII-1	GIII	ALICAR	1-15-2013
GIII-2	GIII	ALICAR	3-20-2013
GIV-1	GIV	ALICAR	1-15-2013

^a^ ALICAR: Alisal Creek inlet to Carr Lake, Salinas, CA, GPS co-ordinates: 36.67736553N -121.63945327W; RECVIC: Reclamation ditch at Victor street, Salinas, CA; GPS co-ordinates:.68406599N, -121.66718732W [14].

^b^ FRNA were isolated from waters sampled on these dates.

### Persistence of prototype strains in surface water

Triplicate 250 mL Erlenmeyer flasks containing 50 mL surface water samples were inoculated with 500 μL portions of prototype FRNA and *E*. *coli* F_amp_ host cells_._ Initial concentrations for FRNA and host cells in each treatment were targeted to be between 6 to 7 log PFU or CFU/mL, respectively. The water was brought to room temperature prior to inoculations and the flasks were kept stationary and in the dark during incubation. Populations of FRNA were monitored for 30 days at two different temperatures and in the presence or absence of the bacterial host. Controls without added FRNA or bacterial host were monitored also to determine the persistence of background populations of FRNA. Survival of each prototype FRNA was monitored at 10°C and 25°C to represent summer and winter temperatures in the Salinas valley region. Average monthly mean temperatures during the past ten years from November to February (winter) was 11.2 ± 0.9°C, March to June (spring) was 14.4 ± 0.6°C and between June to September (summer) was 23.5 ± 2.5°C. The historical data was obtained from the weather station located near KSNS Salinas Municipal Airport, Salinas, CA (http://www.weatherunderground.com/history).

FRNA were enumerated from ten-fold serial dilutions of 100 μL sub-samples of treated and untreated waters at various intervals during the incubation. Double agar layer (DAL) method [25] was used to enumerate FRNA from dilutions prepared in sterile 0.01M phosphate-buffered saline (pH 7.2). Briefly, 100 μL of each of serial dilution of waters and log-phase (4 h growth) *E*. *coli* F_amp_ host cells were added to 5 mL of 0.7% tryptic soy agar (TSA) with ampicillin/streptomycin at 45°C, mixed thoroughly and added on to the top of 1.5% TSA plates with ampicillin/streptomycin. The soft agar was allowed to set and the inverted plates were incubated for 18 h at 37°C for plaque formation. The plaques were counted and D-values (days for one log reduction) were calculated based on the log PFU decline from day zero to the last day on which measurable phage growth was observed. D-values were calculated for each replicate separately, and the significant differences in persistence of prototypes of FRNA at two different temperatures and in the presence or absence of *E*. *coli* host were determined using three way analysis of variance (SigmaPlot 13, Systat Software, Inc., San Jose, CA).

### Persistence of environmental strains in surface water

Survival of native FRNA (Table 2) strains, previously isolated from waters from the same geographical region, was monitored in freshly collected surface water incubated at 25°C. The influence of added *E*. *coli* F_amp_ host was also compared during the stationary incubations for 30 d in the dark. The treatments were in triplicate and populations of environmental FRNA were monitored at various intervals from 100 μL serial dilutions in 0.01M phosphate-buffered saline as described above. Data on D-values of environmental and prototype FRNA in the presence or absence of *E*. *coli* F_amp_ host was analyzed to determine the differences in persistence of FRNA using two-way analysis of variance (SigmaPlot 13).

## Results

### Water chemistry

Since freshly collected waters were used for determining the persistence of each prototype strain of FRNA, waters were analyzed to find out if differences in chemical/physical composition alter phage survival. The chemical composition was similar in waters collected on all occasions, except for an elevated level of suspended solids along with increased turbidity for water collected on May 26, 2015 (Table 1). Waters were alkaline. Survival of each prototype phage was monitored using water collected on separate days and all environmental strains were assayed using water collected on one day. FRNA were not detected in these waters prior to inoculations.

### Persistence of prototype strains of FRNA

The survival of prototype strains of FRNA was monitored in freshly collected surface water to determine the differences in persistence at temperatures representing the summer and winter conditions in the central coast of California. An initial concentration of 6.4 ± 0.2 PFU/mL was measured from waters treated with MS2, GA or QB. The concentration of SP was somewhat higher at 7.5 ± 0.3 PFU/mL. These high concentrations for FRNA were chosen to obtain several experimental points to aid in calculating D-values. The treatments supplemented with the host received 6.4 ± 0.2 CFU/mL of log phase growth of *E*. *coli* F_amp._

**Fig 1:**
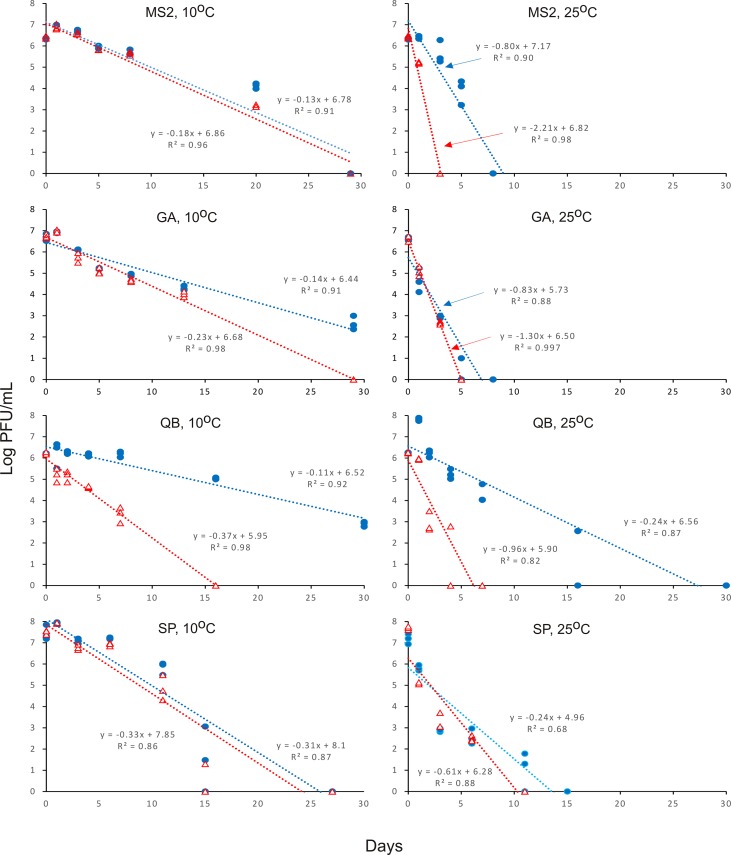
Survival of prototype strains of FRNA in surface waters. FRNA populations of four different genogroups were monitored at two temperatures and in the presence (filled blue circles) or absence of (open red triangles) the host *E*. *coli* F_amp_. Data on survival of prototypes in surface water samples is shown in S1 File.

An increase in phage growth of 0.4 to 0.7 log PFU/mL was observed within one day of incubation at 10°C in treatments of MS2, GA and SP supplemented with the host (Fig 1). A substantially higher increase in growth of 1.5 log PFU/mL on day one was observed with QB plus host at 25°C. Host cells disappeared rapidly with D-values of 5.9 d and 4.0 d at 10°C and 25°C, respectively, from FRNA-free waters.

In general, phages disappeared linearly with time and survived longer at 10°C compared to 25°C (Fig 1). In the absence of the host, they all disappeared in less than 10 d from waters at 25°C. MS2 disappeared more rapidly in <3 d. With the host, both GA and QB survived longer at 10°C and nearly 3 log PFU/mL remained after 30 days. QB survived longer with the host at both temperatures.

D-values were calculated based on phage levels on day zero and the initial growth spurts on day one were ignored. Three-way analysis of variance revealed significant differences (*P* < 0.001) in persistence between prototype strains, in the presence or absence of the host and at different temperatures of incubation (Table 3). In general, FRNA persisted significantly longer in the presence of *E*. *coli* F_amp_ compared to no host and at 10°C compared to 25°C. In the presence of the host, the order of persistence at 25°C was QB>MS2>SP>GA; without the host, prototype strains disappeared rapidly. SP survived significantly longer in the absence of host compared to other prototypes. However, the survival spectrum changed at 10°C and MS2 persisted longer both in the presence or absence of the host and the order of persistence without the host was MS2>GA>SP>QB, whereas the order of persistence with the host was MS2 = QB >GA>SP.

**Table 3:** Influence of temperature of incubation and presence of the host *E*. *coli* F_amp_ on the persistence of prototype strains of FRNA in surface water.

		D-value, d^a^			
Prototype strain	Genogroup	10°C___________		25°C____________	
		No host	Host	No host	Host
MS2	GI	6.21 c	9.05 a	0.83 hi	2.11 f
GA	GII	4.94 d	7.20 b	0.77 hi	0.81 hi
QB	GIII	2.46 f	9.09 a	0.62 i	3.95 e
SP	GIV	3.94 e	4.05 e	1.15 gh	1.46 g

^a^ Three-way analysis of variance statistics: SEM 0.415; *P*<0.001 for strains, temperature of incubation and presence or absence of the host; P = 0.049 for interactions between these factors; F-values, F_strains_ = 15.4; F_temperature_ = 450; F_host_ = 102. Numbers followed by the same letters are not significantly different from each other. Statistical analysis of D-values is shown in S3 File.

### Persistence of environmental strains

**Fig 2:**
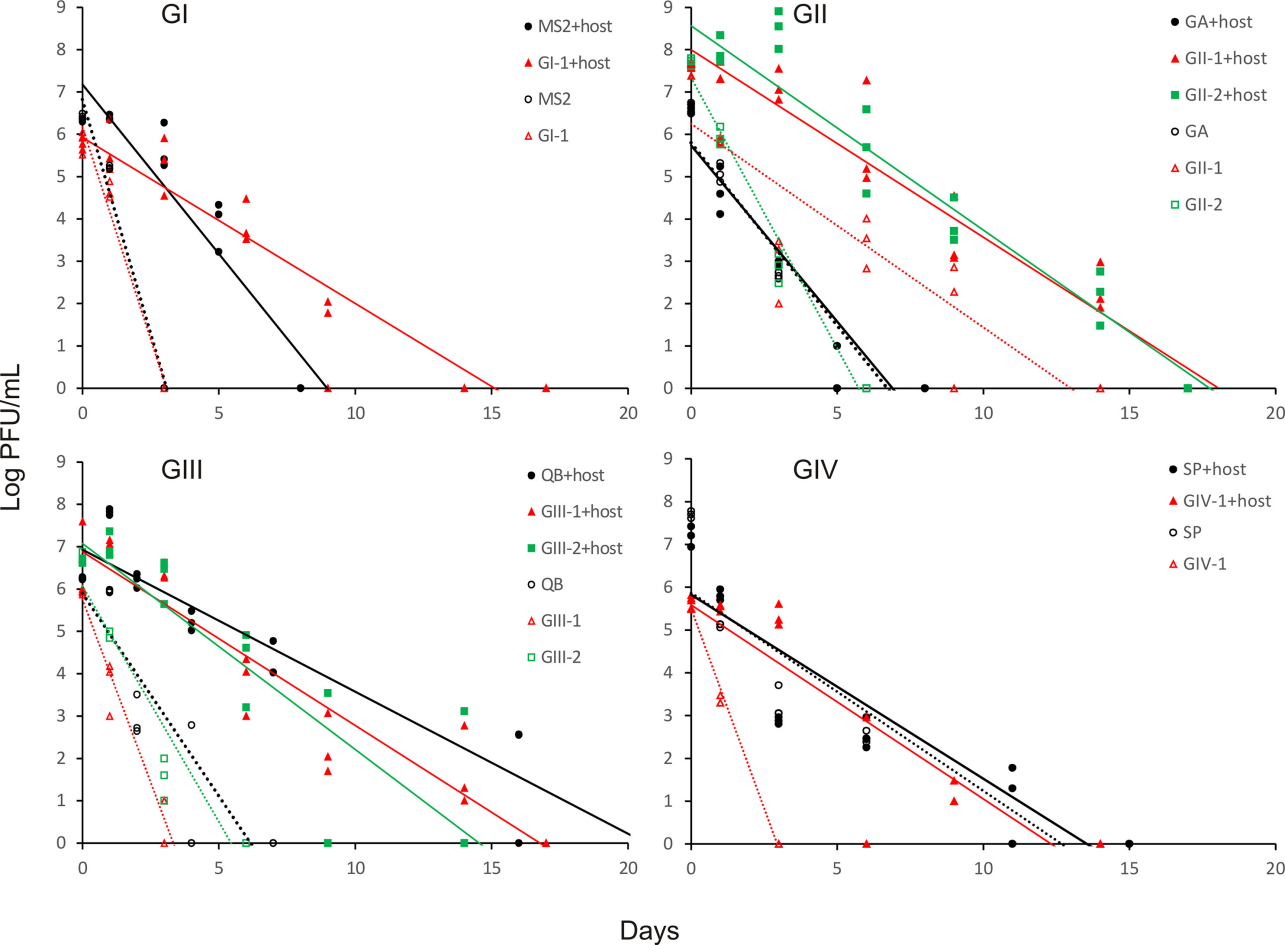
Persistence of environmental and prototype strains of four different genogroups of FRNA in surface waters. The survival of phages was monitored at 25°C and in the presence (filled symbols) or absence (open symbols) of the host *E*. *coli* F_amp._ Phage persistence for prototype strains are shown in black. Regression equations are shown in S1 Table. Data on survival of environmental strains in surface water samples is shown in S2 File.

The survival of environmental strains of FRNA isolated from surface waters at different locations and time periods (Table 2) was compared with that of prototype strains incubated at 25°C. Similar to prototypes, an initial surge in growth was observed in the presence of the host with environmental strains of GII and GIII. Absence of the host generally resulted in rapid disappearance of all phages in <10 d (Fig 2) and the environmental strain GI-1 behaved similar to MS2 by rapidly disappearing in <3 d. With the host, environmental strains of GII and GIII survived similarly or longer than their respective prototypes; these were detected even after 14 d.

Significant differences (*P*< 0.001) in D-values were observed between environmental and prototype strains in the presence or absence of the host. Environmental strains of GI, GII and GIV persisted longer compared to prototypes in treatments amended with the host (Table 4). However, environmental strains GIII-1 and GIII-2 disappeared somewhat faster compared to QB. With the exception of GII-1, absence of the host resulted in rapid disappearance of all environmental strains from surface water samples. In the presence of the host, however, significant differences in persistence of strains were found and the order of persistence was GIII-1 = GIII-2 = GI-1>GII-1 = GII-2>GIV-1.

**Table 4:** Persistence of prototype and environmental strains of FRNA as influenced by the presence of the host *E*. *coli* F_amp_ in surface water incubated at 25°C.

		D-value, d^a^	
Strain	Genogroup	No host	Host
MS2	GI	**0.83** ghij	**2.11** d
GI-1	GI	0.87 ghi	3.25 b
GA	GII	**0.77** ij	**0.81** hij
GII-1	GII	1.60 e	2.72 c
GII-2	GII	0.61 ijk	2.59 c
QB	GIII	**0.62** ijk	**3.95** a
GIII-1	GIII	0.52 ijk	3.19 b
GIII-2	GIII	0.58 ijk	2.88 bc
SP	GIV	**1.15** fg	**1.46** ef
GIV-1	GIV	0.43 k	2.05 d

^a^ Numbers in bold face are D-values for prototype strains. Numbers followed by the same letters are not significantly different from each other. Two way analysis of variance statistics: SEM = 0.330; D-values are significantly different (*P*<0.001) between strains, and presence or absence of *E*. *coli* host; There is statistically significant interaction between strains and host levels; F-values: F_strains_ = 3.963, F_host_ = 133 and F_interaction_ = 4.96. Statistical analysis of D-values is shown in S3 File.

## Discussion

Source tracking of foodborne pathogens is critical for development of pathogen control measures at the source. Water is an obvious source of transport of pathogens from animal raising operations, if they are close to waterways. Indeed, numerous *E*. *coli* O157:H7 and other shiga-toxigenic *E*. *coli* were isolated from surface waters from the produce production region of the central coast of California [26–28]. Since the levels of these pathogens are transient, they are often undetectable during labor-intensive and expensive trace-back studies in the environment. Detection of FRNA as fecal source indicators holds promise, since the contamination of waters can presumably be sourced to animals or humans. However, the differences in persistence of environmental phages make it difficult to distinguish the proportion of FRNA that can be sourced to animals or humans. In addition, seasonal and environmental factors play a significant role in the environmental persistence of FRNA.

### Persistence at 25°C

Summers in Salinas valley don’t appear conducive for the growth of FRNA in surface waters. Both prototype and environmental strains disappeared rapidly with D-values <1.6 d and their infectivity was lost in <10 d. Such rapid inactivation was also observed with prototypes in ground, river and sea waters tested at similar temperatures [11, 21, 22, 29, 30]. To aid the comparison of these published results, D-values were calculated from the reported decay rates. However, others have reported decay rates that give a D-value of >15 d (calculated) for MS2 in lake, mineral and sea waters; and the persistence for prototypes and environmental strains were in the order of GI>GII>GIII>GIV [18–20].The current study differs in that the longest surviving phage is an environmental strain GII-1 (GA-like) with a D-value of 1.6 d and the longest surviving prototype is SP of GIV with a D-value of 1.2 (Table 4). We speculate that the absence of host bacterium resulted in rapid disappearance of phages. Since the inactivation of all phages was rapid, the infectivity assays can detect only recent fecal contamination during the summers in California. Such rapid inactivation, as the waters warm up during the spring, could be a reason for the sparse detection of FRNA after April [14]. Similarly, higher water temperatures were linked with rapid inactivation of FRNA [19, 31], resulting in their infrequent detection from surface waters [32].

### Persistence at 10°C

Cooler temperatures were responsible for high prevalence of FRNA in waters during the months of January to March in Salinas valley [14] and in Massachusetts bay [33]. Thus, prototypes were evaluated for survival at 10°C and were observed to survive significantly longer compared to 25°C (Table 3). In the absence of the host, significant differences in survival between prototypes were observed at 10°C. MS2 and GA were more persistent compared to SP and QB. The order of persistence is comparable to results obtained by others at higher temperatures [18–20]. However, in a separate study at 15°C in river water, no such differences in persistence between genogroups were found [21]. Thus, fluctuations in temperatures influence the persistence and distribution of coliphages in surface waters. Consequently, elevated levels of phages might not always be from recent fecal inputs but could be more indicative of their environmental persistence.

### Persistence in the presence of host bacterium

‘Bacteriophages (phages) are the most abundant replicating entities on the planet and thrive wherever their bacterial hosts exist’ [34]. Indeed, *E*. *coli* strains sensitive to infection by FRNA were commonly found in human and animal feces [35], and a significant correlation was observed between the prevalence of FRNA and *E*. *coli* in tropical surface waters [36]. Accordingly, this study compared the survival of prototype and environmental strains of FRNA in the presence of the host *E*. *coli* F_amp._

FRNA survived significantly longer (*P*<0.001) in the presence of the host and the persistence spectrum of the phage types differed as compared to no host. Prototypes QB followed by MS2 persisted longer compared to the other prototypes incubated at either 10°C or 25°C (Table 3). Similarly, QB-like (GIII-1 and GIII-2) and MS2-like (GI-1) phages persisted with the host compared to other environmental phages. To the best of our knowledge, there are no other reports that evaluated the significance of the presence of the host on FRNA survival in the environment, although both phage and host are released together in the feces of animals and humans. Determining host prevalence along with FRNA may aid in improved prediction of fecal sources, since the presence of *E*. *coli* was positively correlated with FRNA [36] while its absence resulted in the rapid disappearance of coliphages from surface waters.

Host presence also caused growth of FRNA. This is predictable as the waters treated with the host received 6.4 log CFU/mL of *E*. *coli* F_amp._ This resulted in replication of phages for 1 to 3 d (Figs 1 and 2). Further phage growth was likely hampered as the host populations decreased with D-values of 4 to 6 d. It was reported that QB failed to replicate when host cells were fewer than 4 log CFU/mL [37]. However, using QB as a model, it was also reported that replication ceases below 25°C [38]. In contrast, this study observed growth with 3 out of 4 prototypes in the presence of the host within a day of incubation at 10°C. Availability of host bacterium significantly influences the survival, growth and prevalence of FRNA in surface waters.

### Persistence of environmental strains

Significant differences in survival were observed between prototype and environmental strains of FRNA. A majority of the environmental strains persisted longer than the prototypes when the host was present and strain differences in survival were observed between genogroups. Similar strain differences in persistence were also reported for other environmental isolates [19, 24]. While a majority of the environmental strains disappeared rapidly in the absence of added host, members of GIII and GI followed by GII persisted longer with the host. This is consistent with our earlier, preferential isolation of members of GIII followed by GII and GI from surface waters from the same geographical region [14]. Therefore, survival of environmental and prototype strains of FRNA varies within and between genogroups and is influenced by prevalence of hosts native to the environment.

### Persistence and water chemistry

Differences in the chemical constituents of waters did not influence the persistence of prototype strains. The absence of effect is evident as the differences in D-values, at 25°C without the host, between MS2, GA and QB were not significantly different from each other and so as the differences between MS2, GA and SP (Table 3). This might have been predictable as salt concentrations of up to 42 g/L in artificial sea water failed to alter the survival of FRNA compared to mineral water [18]. In addition, it was reported that suspended solids have a protective effect on the survival of viruses [39], while removal of solids results in rapid inactivation [21]. However, in the present study, relatively high amount of solids had no effect, as QB was inactivated at 25°C as rapidly as the other phages incubated in waters with less solids (Tables 1 and 3). Furthermore, FRNA were reported to persist longer as pH decreases from 8.4 to 5.5 [21], and the higher pH (8.1) of surface waters in this study might have aided the rapid disappearance of all FRNA.

## Conclusions

Significant differences exist in the persistence of environmental and prototype strains of FRNA. Persistence fluctuated with seasonal temperatures and the availability of bacterial host. Therefore, source tracking attempts would likely detect phages from recent fecal inputs compared to those that persist under fluctuating environmental conditions. Future studies that expand to different geographic locations and include infectivity assays with natural hosts may aid in determining an accurate assessment of prevalence of FRNA in surface waters. Since other fecal indicators (*E*. *coli*, enterococi, *Bacteroides thetaiotaomicron* and others) fluctuate similarly as FRNA [36], multiple indicators may be chosen for improved source tracking of fecal inputs to develop strategies to limit the hazards of fecal contamination of ground and surface water.

## Supporting Information

S1 File Survival of prototype strains of FRNA in surface water samples. Excel 2007 file.
(XLSX)

S2 File Survival of environmental strains of FRNA in surface water samples. Excel 2007 file.
(XLSX)

S3 File Statistical analysis of D-values for prototype and environmental strains of FRNA. SigmaPlot 13 notebook.
(JNB)

S1 Table Linear regression equations corresponding to survival plots in Fig 2. (DOCX)
