# Supplementary material for: Persistence of F-Specific RNA Coliphages in Surface Waters from a Produce Production Region along the Central Coast of California
Source: PLoS One. 2016 Jan 19;11(1):e0146623. doi: 10.1371/journal.pone.0146623 (PMC4718509; doi:10.1371/journal.pone.0146623)
Supplement: S1 Table — (DOCX) [file pone.0146623.s004.docx]

S1 Table. Linear regression equations corresponding to survival plots in Fig 2.

|  |  |  |  |  |  |
| --- | --- | --- | --- | --- | --- |
|  |  | No host |  | Host |  |
|  |  | Linear regression | R2 | Linear regression | R2 |
| MS2 | GI | Y = -2.21x+6.82 | 0.98 | Y=-0.80x+7.17 | 0.90 |
| GI-1 | GI | Y = -2.0x+6.17 | 0.98 | Y=-0.40x+6.10 | 0.88 |
| GA | GII | Y=-1.30x+6.50 | 0.997 | Y=-0.83x+5.73 | 0.88 |
| GII-1 | GII | Y=-0.48x+6.25 | 0.8 | Y=-0.44x+8.00 | 0.94 |
| GII-2 | GII | Y=-1.28x+7.34 | 0.98 | Y=-0.48x+8.56 | 0.93 |
| QB | GIII | Y=-0.96x+5.90 | 0.82 | Y=-0.20x+6.30 | 0.97 |
| GIII-1 | GIII | Y=-1.73x+5.75 | 0.96 | Y=-0.41x+6.88 | 0.91 |
| GIII-2 | GIII | Y=-1.11x+6.07 | 0.92 | Y=-0.49x+7.07 | 0.78 |
| SP | GIV | Y=-0.61x+6.28 | 0.88 | Y=-0.24x+4.96 | 0.68 |
| GIV-1 | GIV | Y=-1.86x+5.50 | 0.99 | Y=-0.45x+5.59 | 0.79 |
